# Establishment of a Highly Efficient In Vitro Regeneration System for *Nandina domestica* ‘Firepower’

**DOI:** 10.3390/plants14030421

**Published:** 2025-01-31

**Authors:** Xin Zhao, Hao Dong, Yanhua Li, Xinxin Zhang, Yajing Ning, Chengpeng Cui, Shujuan Li

**Affiliations:** 1 State Key Laboratory of Tree Genetics and Breeding, Northeast Forestry University, Harbin 150040, China; zhxin@nefu.edu.cn (X.Z.);; 2 Zhejiang Physical and Chemical Technology Co., Ltd., Hangzhou 310000, China

**Keywords:** *Nandina domestica* ‘Firepower’, plant tissue culture, garden ornamental plants, rapid propagation

## Abstract

*Nandina domestica* ‘Firepower’ is one of the most popular colorful foliage species in landscaping. However, it is currently propagated mainly by seeding and cuttings, with a low reproduction coefficient, hindering the cultivation of this species. Therefore, establishing an in vitro regeneration system would be beneficial for the industrialized production of *Nandina domestica* ‘Firepower’. In this study, an ex vivo regeneration system was established using the direct organogenesis pathway. In early April, the new shoots of *Nandina domestica* ‘Firepower’ were selected, and the stem segments of 1~2 cm were cut as the disinfection materials for the explants. The optimal formulation for inducing axillary shoots was 1/2 MS + 1.5 mg L^−1^ 6-benzylaminopurine (BA) + 0.3 mg L^−1^ indole-3-butric acid (IBA). The optimal formulation for the differentiation and proliferation of axillary shoots was 1/2 MS + 1.5 mg L^−1^ BA + 0.01 mg L^−1^ IBA with a multiplicity of proliferation of 9.22. We determined that the rooting of axillary shoots required a combination of IBA, naphthalene acetic acid (NAA), and activated carbon (AC). The optimal formulation for rooting was 1/2 MS + 0.2 mg L^−1^ NAA + 0.3 mg L^−1^ IBA + 0.2 mg L^−1^ AC. After a two-day hardening period for tissue-cultured plantlets, a substrate consisting of peat soil, vermiculite, and perlite at a ratio of 2:2:1 was determined to be the optimal cultivation formulation. This system provides a framework for the industrialized production of *Nandina domestica* ‘Firepower’.

## 1. Introduction

*Nandina domestica* ‘Firepower’ is a cultivated variety of *Nandina domestica* Thunb in the Berberidaceae family and the genus *Nandina*. It is an evergreen shrub with dark green leaves that often display a red hue during the winter months. The species Nandina domestica is indigenous to North China, Central China, East China, and parts of South China. Additionally, the plant exhibits a number of beneficial characteristics, including drought resistance, wind resistance, high-temperature resistance, cold resistance, snow pressure resistance, and strong resistance to pests and diseases. Presently, *Nandina domestica* Thunb in China is primarily concentrated in Jiangsu, Zhejiang, Anhui, Jiangxi, Hunan, Hubei, Sichuan, Shaanxi, and Hebei, with a notable presence in various horticultural settings [1,2]. *Nandina domestica* ‘Firepower’ thrives in a warm, moist, well-ventilated, semi-shaded environment, preferring sandy loams rich in humus [3]. *Nandina domestica* ‘Firepower’ is a short plant with short nodes [4]. As temperatures decline in autumn, the foliage undergoes a transformation, shifting from its verdant hue to a vibrant red. This striking seasonal change can persist until the following spring, offering a compelling ornamental quality [5]. As society progresses and living standards improve, people have increasingly high expectations of the quality of the environment, particularly with regard to plants. The public is becoming increasingly interested in plants of different colors, leading to the development of some colorful varieties [6]. *Nandina domestica* ‘Firepower’ is used in garden landscapes such as flower beds and flower mirrors for its compact growth and the fact that its leaves turn red leaves in winter, providing vibrant color for winter scenery [7].

Nandina domestica ’Firepower’ can be propagated through two methods: sexual reproduction (seeding) and asexual reproduction (ramet division and cutting). However, both methods exhibit low propagation efficiencies and extended growth cycles, making them susceptible to environmental influences. Specifically, the germination rate of seeding is only 60% to 70%, and it takes 2 to 3 years for seedlings to develop into mature plants suitable for landscaping or ornamental purposes. Ramet division is typically performed every 3 to 4 years; excessive division can negatively affect plant growth and fruiting. The rooting success rate of cuttings is approximately 70% to 80%, with strict requirements for environmental conditions such as humidity and temperature [8,9]. Plant tissue-culture technology offers many advantages, such as high reproduction efficiency, the stable growth of seedlings, and unrestricted reproduction. The use of plant tissue culture can enable the rapid propagation of the *Nandina domestica* ‘Firepower’ to meet market demand. To date, various cultivars of *Nantian bamboo* have been successfully propagated in vitro, including the “Lemon yellow” and “Habo” varieties [10]. In 2007, the first study on the micropropagation of *Nantian bamboo* was conducted, utilizing stem segments from that year’s shoots as explants to identify the optimal medium for germination, redifferentiation, and rooting [11]. In 2010, Li Hui developed an in vitro culture system using sterilized vaccine stem segments as explants, investigating the impacts of different basal media, plant growth regulators, and activated carbon on plant regeneration [5]. However, in that in vitro culture system, the proliferation efficiency of the adventitious buds was low.

Therefore, with the aim of attaining higher reproductive efficiency, a stable and efficient in vitro culture system was developed on the basis of vigorous-growing and pest- and disease-free materials.

## 2. Results

### 2.1. Basal Medium Selection

**Figure 1:**
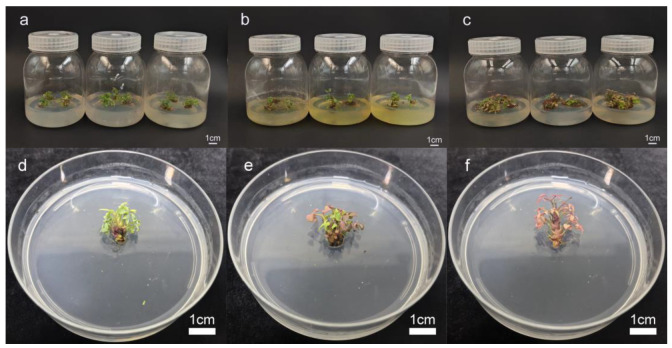
Effect of medium type on axillary shoots’ initiation. (**a**) 1/2MS; (**b**) MS; (**c**) WPM. (**d**) The culture was maintained in a 1/2 MS medium for a period of 30 days. (**e**) The culture was maintained in a MS medium for a period of 30 days. (**f**) The culture was maintained in a WPM medium for a period of 30 days.

As shown in Table 1 and Figure 1, the type of medium had a significant effect on the formation and growth of axillary shoots in *Nandina domestica* ‘Firepower’. When inoculated to the 1/2 Murashige and Skoog (1/2 MS) medium, the axillary shoots’ leaves were tender and green with robust growth (Figure 1a,d); when inoculated with Murashige and Skoog (MS) medium, the axillary shoots’ leaves were tender and green, but the leaf surface was curled, the base was browned, and the medium was yellowed (Figure 1b,e); and when inoculated with woody plant medium (WPM), the axillary shoots leaves were yellowed, and growth was wilted (Figure 1c), (Figure 1f). Therefore, considering the induction of axillary shoots and plant growth, 1/2 MS was chosen as the optimal basal medium in this experiment.

### 2.2. Induction of Axillary Shoots from Tender Stem

As shown in Table 2, the concentrations of 6-benzylaminopurine (BA) and indole-3-butyric acid solution (IBA) had significant effects on the induction of axillary shoots in *Nandina domestica* ‘Firepower’. When the concentration of IBA was 0.5 mg-L^−1^, the time required for inducing the axillary shoots was gradually shortened with the increase in the BA concentration. When the concentration of IBA was increased, the time required for inducing axillary shoots became longer at the same concentration of BA. Comparing the growth of axillary shoots and the time required to induce axillary shoots, we found that high concentrations of IBA inhibited the induction of axillary shoots in *Nandina domestica* ‘Firepower’; therefore, after a comprehensive comparison, 1.5 mg-L^−1^ BA and 0.3 mg-L^−1^ IBA were found to be the optimal choices for axillary shoots’ induction.

### 2.3. Proliferation of Axillary Shoots

**Figure 2:**
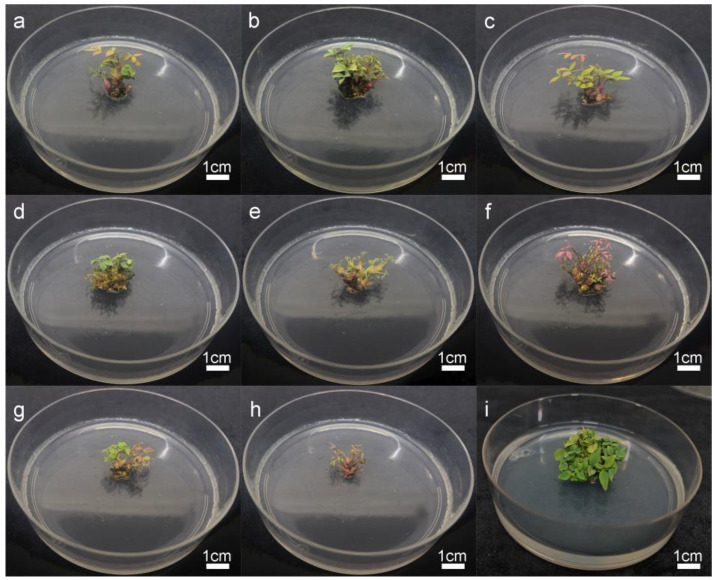
Effect of BA and IBA concentrations on shoot proliferation: (**a**) 1 mg-L^−1^ BA + 0.01 mg-L^−1^ IBA; (**b**) 1 mg-L^−1^ BA 0.03 mg-L^−1^ IBA; (**c**) 1 mg-L^−1^ BA 0.05 mg-L^−1^ IBA; (**d**) 1.5 mg-L^−1^ BA + 0.01 mg-L^−1^ IBA; (**e**) 1.5 mg-L^−1^ BA + 0.03 mg-L^−1^ IBA; (**f**) 1.5 mg-L^−1^ BA + 0.05 mg-L^−1^ IBA; (**g**) 1.5 mg-L^−1^ BA + 0.1 mg-L^−1^ IBA; (**h**) 1.5 mg-L^−1^ BA + 0.3 mg-L^−1^ IBA. (**i**) Subsequent cultures were cultivated on two occasions at a concentration of 1.5 mg L^−1^ BA and 0.01 mg L^−1^ IBA hormones.

As shown in Table 3 and Figure 2, the concentrations of BA and IBA had significant effects on the proliferation of axillary shoots. When the concentration of IBA was constant, upon increasing the concentration of BA, the proliferation rate of axillary shoots of tended to increase; when the concentration of BA was constant, with the increase in the concentration of IBA, the proliferation rate of the axillary shoots tended to decrease. A comprehensive analysis of the proliferation rate and growth state of the axillary shoots revealed that the optimal medium for axillary shoots proliferation was 1/2 MS + 1.5 mg-L^−1^ BA + 0.01 mg-L^−1^ IBA, and the proliferation rate for axillary shoots was 9.22.

### 2.4. Induction of Adventitious Roots

**Figure 3:**
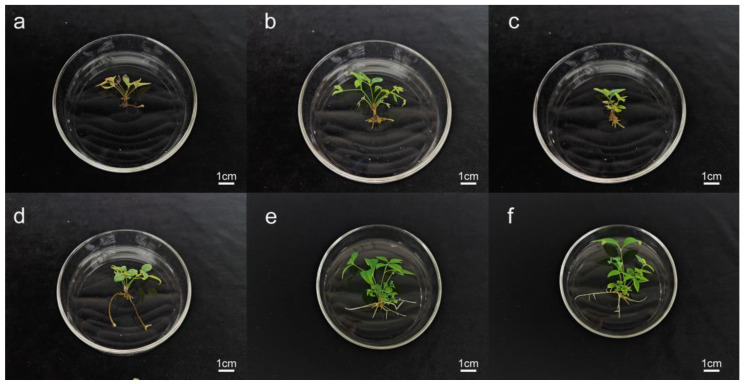
Effect of IBA, NAA, and AC concentrations on adventitious roots: (**a**) 0.2 mg-L^−1^ NAA + 0.4 mg-L^−1^ IBA + 0.4 mg-L^−1^ AC; (**b**) 0.4 mg-L^−1^ NAA + 0.2 mg-L^−1^ IBA + 0.4 mg-L^−1^ AC; (**c**) 0.4 mg-L^−1^ NAA + 0.2 mg-L^−1^ IBA + 0.4 mg-L^−1^ AC; (**d**) 0.4 mg-L^−1^ NAA + 0.3mg-L^−1^ IBA + 0.5 mg-L^−1^ AC; (**e**) 0.3 mg-L^−1^ NAA + 0.2 mg-L^−1^ IBA + 0.2 mg-L^−1^ AC; (**f**) 0.2 mg-L^−1^ NAA + 0.3 mg-L^−1^ IBA + 0.2 mg-L^−1^ AC.

The impact of varying concentrations of naphthalene acetic acid (NAA), indole-3-butyric acid (IBA), and activated carbon (AC) on adventitious root growth was evaluated using the metrics presented in Table 4 and the data illustrated in Figure 3. The adventitious roots of tissue-cultured microshoots were primarily situated within the medium, with secondary roots becoming visible after approximately 20 days. Preliminary experiments indicated that the individual application of NAA or IBA to the medium led to a decrease in the number of roots formed, suggesting that these two plant growth regulators must act synergistically. When the concentration of NAA was held constant, an increase in the IBA concentration inhibited the development of adventitious roots. Conversely, at a fixed IBA concentration, the addition of low concentrations of NAA promoted the formation of adventitious roots. Taking into account the rooting rate, the number of roots, and their growth condition, the optimal rooting formula was determined to be 1/2 MS supplemented with 0.2 mg·L^−1^ NAA, 0.3 mg·L^−1^ IBA, and 0.2 mg·L^−1^ AC, achieving a rooting rate of 88.67% and an average of 6.65 roots per plant.

### 2.5. Acclimatization

**Figure 4:**
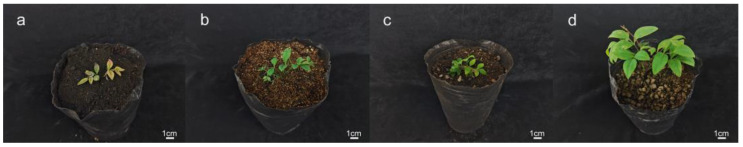
Growth of *Nandina domestica* ’Firepower’ in different substrates: (**a**) peat soil; (**b**) peat soil: vermiculite = 1:1; (**c**) peat soil: vermiculite: perlite = 2:2:1; and (**d**) after 60 days incubation in a mixed substrate of peat soil: vermiculite: perlite of 2:2:1.

In this experiment, the impact of various substrates on the acclimatization of *Nandina domestica* ‘Firepower’ tissue-culture plantlets was investigated. Following a two-day acclimation period, the tissue-culture plantlets were transplanted into substrates composed of differing ratios of vermiculite, peat, and perlite. As shown in Table 5 and Figure 4, the plantlets initially cultivated in peat grew vigorously. However, as the cultivation time extended, there was a tendency for the edges of the leaves to dry out. After 30 days, the survival rate was only 3%. The plantlets cultivated in a 1:1 mixture of peat and vermiculite showed weak and slow growth after transplantation, and their survival rate was 26% after 30 days. In contrast, the plantlets grown in a 2:2:1 mixture of peat, vermiculite and perlite were vigorous and well-developed, with a survival rate of 86% after 30 days.

## 3. Discussion

*Nandina domestica* ‘Firepower’ is a shrub grown in gardens. It is typically planted in clusters or as a standalone specimen in flower beds and courtyards. Additionally, it can be utilized as ground cover and as a colorful accent plant. It is a highly sought-after material for flower beds and courtyards. Market demand for this plant is considerable [12]. The propagation of *Nandina domestica* ‘Firepower’ is typically achieved through dividing and cutting. However, this method is not without limitations. These include a relatively low propagation coefficient, the potential for the species characteristics to be compromised, and a lengthy cultivation period [13]. In recent years, the increasing demand for *Nandina domestica* ‘Firepower’ has rendered the traditional propagation method inadequate to meet market demand [11]. Nevertheless, the in vitro propagation of plant species could yield a considerable number of robust plantlets with stable growth patterns in a relatively brief period of time. In this study, we established a comprehensive system of plant tissue culture, encompassing axillary shoots’ induction, shoot cluster induction, rooting, and transplantation. The choice of medium and the concentration of plant growth regulators have important effects on the induction and proliferation of axillary shoots in *Nandina domestica* ‘Firepower’. According to Liu Zhong bing et al., the optimal combination for inducing axillary shoots of *Nandina domestica* ‘Firepower’ is MS + 1.0 mg-L^−1^ 6 BA + 0.1 mg-L^−1^ NAA + 20 g-L^−1^ sucrose, which gave the highest induction rate of 74.4% [14]. Deng Yuying et al. observed that vitrification occurred in histo-cultured axillary shoots when the BA concentration reached 0.8 mg-L^−1^. It is, therefore, thought that the concentration of BA in the culture medium should not be excessive [15]. However, our results show that the optimal medium for inducing axillary shoots of *Nandina domestica* ‘Firepower’ was 1/2 MS + 1.5 mg/L BA + 0.3 mg/L IBA. The induction rate could reach 100%, and the generation time of axillary shoots was relatively short. Axillary shoots could be observed after 7 days of culture. Furthermore, high concentrations of BA were more conducive to the formation of axillary shoots. The discrepancy in the observed outcomes may be attributed to the fact that different varieties of nandina may necessitate varying concentrations of BA for optimal growth. Zhou Zhi jiang et al. demonstrated that the optimal proliferation medium for *Nandina domestica* ‘Firepower’ was MS + 1.0 mg-L^−1^ BA + 0.1 mg-L^−1^ NAA + 30 g-L^−1^ sucrose, with a proliferation coefficient of 3.5 observed [16]. In a study of rapid propagation technology of *Nandina domestica* ‘Firepower’, Du Yong qin et al. demonstrated that MS + 1.0 mg of L^−1^ BA + 0.1 mg-L^−1^ IBA was the optimal proliferation medium, exhibiting the highest value-added coefficient of 4.12. In a separate study, Song Shuai jie et al. used young stem tips as the research object. They found that the optimal proliferation medium was MS + 1.0 mg-L^−1^ BA + 0.1 mg-L^−1^ NAA + 0.1 mg-L^−1^ IAA + 1.0 g-L^−1^ AC, which yielded the highest proliferation coefficient of up to 5.29 [17]. Our results show that the optimal medium for the proliferation of axillary shoots of *Nandina domestica* ‘Firepower’ was 1/2 MS + 1.5 mg/L BA + 0.01 mg/L IBA, with a proliferation coefficient as high as 9.22, which was significantly higher than the results of other studies on the proliferation of axillary shoots of *Nandina domestica* ‘Firepower’. The observed variability in results may be attributed to the differing hormone types and ratios, which exert some influence on the growth of late-stage histo-cultured microshoots. In a rooting test conducted by Wang Chun et al. on bamboos (Nandina), the addition of IBA at a concentration of 0.25 mg L-1 resulted in a rooting rate of up to 87.33% [18]. Cai Peng et al. demonstrated that the rooting rate was 76.67% in a liquid medium comprising 1/2 MS + 0.2 mg L^−1^ NAA + 0.2 mg L^−1^ IBA + 4 g L^−1^ AC [19]. We explored the influences of NAA, IBA, and AC on the rooting of micro-branches and the strengthening of microshoots. The results indicated that the combination of 1/2 MS + 0.2 mg/L NAA + 0.3 mg/L IBA + 0.2 g/L AC significantly enhanced the rooting rate of *Nandina domestica* ‘Firepower’, reaching up to 88%. The difficulties associated with rooting and the low survival rate of transplants represent significant challenges in the field of woody tissue-culture breeding. These obstacles impede the implementation and advancement of tissue-culture breeding technology in industrial production settings [20,21]. The addition of AC to the culture medium adsorbs harmful metabolites and promotes the generation of adventitious roots [22]. With regard to the transplantation of plantlets, Song Shuai jie and colleagues have demonstrated that the survival rate of transplanted plants can reach 94% when the transplantation substrate is a mixture of peat soil, vermiculite, and perlite at an 8:1:1 ratio [17] Zhang Jun lin et al. concluded that a 3:2 mixture of peat soil and perlite was more conducive to the growth and development of B. nantianensis [10]. We compared the influences of several substrate mixtures on the growth of *Nandina domestica* ‘Firepower’. Ultimately, a 2:2:1 mixture of peat soil, vermiculite, and perlite was chosen as the cultivation substrate. Its survival rate can reach 86%. Subsequent studies will further explore the appropriate cultivation conditions for *Nandina domestica* ‘Firepower’.

## 4. Materials and Methods

### 4.1. Plant Materials

*Nandina domestica* ‘Firepower’ was purchased from the Zhejiang flower and seedling market. Seven three-year-old *Nandina domestica* ‘Firepower’ plants were selected as raw materials. In early April, the new shoots of *Nandina domestica* ‘Firepower’ were selected, and the stem segments of 1~2 cm were cut as the main materials for the surface sterilization of explants.

### 4.2. Components of the Medium

The chemicals used were as follows: MS Base Salts with vitamins (PM519); 6-benzylaminopurine (BA) (1 mg·mL^−1^), indole-3-butric acid solution (IBA) (1 mg·mL^−1^), and naphthalene acetic acid (NAA) (1 mg·mL^−1^) were from Phyto Technology Laboratories (Lenexa, KS, USA); sucrose (A502792), AGAR (A505255), and active charcoal (A502167) were from Sengon Biotech (Shanghai, China).

### 4.3. Culture Media and Conditions

In this research, three types of media, namely WPM, MS, and 1/2 MS, were utilized. The sucrose concentration of all these three media was 30 g/L, the agar concentration was 5.4 g/L, and the pH value was 5.8 ± 0.02. The types and concentrations of the plant growth regulators in the media employed at different cultivation stages were diverse. All media were sterilized at 121°C for 20 minutes prior to their utilization. The culture temperature was 25 ± 1 °C, the light intensity was 40–50 μmol·m^−2^·s^−1^, and a light/dark cycle of 16/8 h was used.

### 4.4. Sterilization of Explant

In early April, the new shoots of *Nandina domestica* ‘Firepower’ were harvested, and 1–2 cm long stem segments were excised to serve as explants. They were initially rinsed under running water for 120 min (given that the internodes of *Nandina domestica* ‘Firepower’ are extremely short, a prolonged rinsing period is necessary to eliminate certain external contaminant sources) Subsequently, they were transferred into tissue-culture flasks. All subsequent procedures were conducted in a vertical laminar flow cabinet. The explants were initially surface-sterilized with 75% ethanol for 30 s, followed by rinsing 3–5 times with sterile water to eliminate the residual ethanol. Subsequently, disinfection was continued using 2% sodium hypochlorite for 10 min, after which the explants were rinsed again 3–5 times with sterile water. Eventually, the stem segments were dried on sterile filter paper to prepare for the induction of axillary shoots.

### 4.5. Basal Culture Medium Selection

The stem segments obtained from the sterilized materials in the previous stage were used as the experimental materials. These stem segments were inoculated into three different culture media: WPM, MS, and 1/2MS, and cultured for 30 days to monitor their growth. Each bottle contained three stem segments, with a total of 10 bottles per treatment, and each treatment was replicated three times. After the 30-day culture period, the growth status of the stem segments was evaluated and recorded.

### 4.6. Induction of Axillary Shoots from Tender Stem

Sterilized stem segments were used as experimental materials and transferred to media containing BA (0.5, 1.0, or 1.5 mg L^−1^) and IBA (0.3 or 0.5 mg L^−1^) to induce the formation of axillary shoots. Three to four stem segments were inoculated into each bottle. For each combination, a total of 30 bottles were inoculated (10 bottles per replicate, with three replicates). After a 30-day culture period, the time of emergence and growth status of the axillary shoots were recorded. Detailed information on these combinations is provided in Table 2.

### 4.7. Subculture of Axillary Shoots

The axillary shoots induced in the previous stage were transferred to media containing BA (1.0 or 1.5 mg L^−1^) and IBA (0.01, 0.03, 0.05, 0.1, or 0.3 mg L^−1^) for subculture. Each bottle contained three to four stem segments, with a total of 10 bottles inoculated per treatment, and each treatment was replicated three times. After a 30-day incubation period, the proliferation and growth status of the shoots were carefully observed and recorded. The proliferation coefficient was calculated by dividing the total number of new shoots formed by the number of initially inoculated buds.

### 4.8. Rooting

Microshoots measuring between 1.5 and 2.5 cm in height were meticulously selected, separated from the shoot clusters, and subsequently cultured in a 1/2 MS medium supplemented with varying concentrations of IBA, NAA, and AC. Each bottle contained three stem segments, with a total of 10 bottles inoculated per treatment, and each treatment was replicated three times. After a 30-day culture period, the number, length, and time required for root formation were carefully documented. The rooting efficiency was quantified as the ratio of microshoots that successfully developed roots to the total number of microshoots inoculated into the medium.

### 4.9. Ex Vitro Acclimatization

Plantlets with well-developed roots were selected from the rooting culture for acclimatization. An appropriate amount of distilled water was added to the tissue-culture flasks, which were left open gradually over 1 to 2 days to allow the plantlets to acclimate to the external environment during this period. After acclimatization, the plantlets were carefully removed from the flasks, and their roots were gently washed to remove any residual culture medium. The plantlets were then planted in substrates containing different proportions of vermiculite, peat soil, and perlite. Thirty plantlets were planted in each substrate type (10 plantlets per replicate, with three replicates). After a 30-day growth period, the survival rate and growth conditions of the plantlets were observed and recorded.

### 4.10. Statistical Analysis

The experimental data are presented as the mean ± standard error (SE) and were analyzed through SPSS 25.0 using ANOVA followed by Duncan’s multiple range test. The significance level was set at *p* < 0.05. Tables were constructed via Microsoft Excel 2010 and Microsoft Word 2021, and the figures were generated using PS 2020.

## 5. Conclusions

In this study, we established an in vitro propagation system for *Nandina domestica* ‘Firepower’ via direct organogenesis. In early April, the new shoots of *Nandina domestica* ‘Firepower’ were selected, and the stem segments of 1~2cm were cut as the main materials for the surface sterilization of explants. The optimal protocol for explant sterilization was found to be a two-hour rinse with running water, a 30 s treatment with 75% ethanol, and a 10 min treatment with 2% NaOCl. The optimal medium was identified as 1/2 MS basal medium, and the most effective formulation for the induction of axillary shoots was 1/2 MS medium supplemented with 1.5 mg L^−1^ BA and 0.3 mg L^−1^ IBA. The shoots of tissue-culture seedlings were used to induce axillary shoots, and the optimal concentrations for subculture and proliferation were found to be 1.5 mg L^−1^ BA and 0.01 mg L^−1^ IBA. Rooting was performed using 1/2 MS + 0.2 mg L^−1^ NAA + 0.3 mg L^−1^ IBA + 0.2 mg L^−1^ AC. Finally, the optimal substrate was found to be vermiculite, peat soil and perlite in a 2:2:1 ratio. Nevertheless, it is widely acknowledged that the genotype significantly influences plant regeneration, and certain variations exist among different genotypes. Hence, in future studies, we will conduct research on other genotypes of this plant to enrich the in vitro culture system of this plant.

## Figures and Tables

**Table 1:** Effect of medium types on the growth of axillary shoots.

Group	Type of Medium	Plant Growth
1	1/2 MS	Tender green leaves and robust growth of axillary shoots in tissue culture
2	MS	Tissue-cultured axillary shoots with tender green leaves but with curled leaves and browning at the base.
3	WPM	Yellowish and shriveled leaves of tissue-cultured axillary shoots

**Table 2:** Effect of PGR and concentration on the induction of axillary shoots.

Groups	BA Concentration (mg L ^−1^)	IBA Concentration (mg L ^−1^)	Sprouting Time/Day	Growth State of Buds
1	0.5	0.3	13.4 ± 0.90 a	Leaf blades young and green but slightly curled on the leaf surface
2	1	0.3	9.84 ± 0.65 d	Tender green leaves but foliage curled
3	1.5	0.3	7.08 ± 0.38 e	Tender green leaves and strong growth
4	0.5	0.5	11.6 ± 0.24 c	Leaves slightly yellowed
5	1	0.5	12.36 ± 0.88 b	Yellowing of leaves
6	1.5	0.5	12.48 ± 0.50 b	Basal browning

Means followed by the same letters in rows are not significantly different at *p* ≤ 0.05.

**Table 3:** Effects of different BA and IBA concentrations on the induction of bud clusters.

Groups	BA Concentration (mg L ^−1^)	IBA Concentration (mg L ^−1^)	Multiplication Coefficient	Growth State of Buds
1	1	0.01	3.98 ± 0.38 b	Average growth, curled leaves, few differentiated buds
2	1	0.03	4.00 ± 0.67 b	Many differentiated buds, good growth, thick leaves
3	1	0.05	3.00 ± 0.33 c	Average growth, curled leaves, few differentiated buds
4	1.5	0.01	9.22 ± 0.84 a	Robust growth, normal leaf shape, maximum number of differentiated buds
5	1.5	0.03	4.33 ± 0.57 b	Many differentiated buds, medium growth, browning at base
6	1.5	0.05	2.66 ± 0.66 cd	Few differentiated buds, average growth, yellowing leaf margins
7	1.5	0.1	1.88 ± 0.50 e	Few differentiated buds, with browning at the base
8	1.5	0.3	1.44 ± 0.19 e	Differentiated buds, few in number, brownish at the base, partly vitrified

Means followed by the same letters in rows are not significantly different at *p* ≤ 0.05.

**Table 4:** Effect of different concentrations of NAA, IBA, and AC on the rooting of axillary shoots.

Groups	NAA Concentration (mg L ^−1^)	IBA Concentration (mg L ^−1^)	AC Concentration (mg L ^−1^)	Adventitious Roots Number	Rooting Rate	Grouped Microshoots and Root Growth Status
1	0.2	0.2	0	0.00 ± 0.00 d	0.00%	Microshoots are slow growing, with healing wounds at the base and no root formation.
2	0.2	0.3	0.2	6.65 ± 0.59 a	88.67%	Microshoots are strong, with a large number of roots and secondary root formation.
3	0.2	0.4	0.4	1.66 ± 0.57 bcd	27.67%	Microshoots are poor, with yellowing leaves and weak roots.
4	0.2	0.5	0.5	1.66 ± 1.52 bcd	11.33%	Microshoots are poorly grown, with deformed leaf blades and a low number of roots.
5	0.3	0.2	0.2	5.36 ± 0.37 a	55.67%	Microshoots are robust, with low root formation.
6	0.3	0.3	0	0.00 ± 0.00 d	0.00%	Microshoots are slow growing, with healing wounds at the base and no root production.
7	0.3	0.4	0.5	1.83 ± 1.04 bc	22.33%	Microshoots are strong, with low root production.
8	0.3	0.5	0.4	1.33 ± 1.15 bcd	11.33%	Microshoots are poorly developed, with deformed leaves and weak roots.
9	0.4	0.2	0.4	2.66 ± 0.76 b	33.00%	Microshoots are slow growing, with short roots.
10	0.4	0.3	0.5	1.83 ± 0.76 bc	22.33%	Microshoots are slow to grow, and fewer roots are produced.
11	0.4	0.4	0	1.33 ± 2.30 bcd	5.67%	Microshoots are poor, with healing wounds forming at the base.
12	0.4	0.5	0.2	0.33 ± 0.57 cd	5.67%	Microshoots poorly grown, and few roots are formed.
13	0.5	0.2	0.5	1.00 ± 1.00 bcd	16.67%	Microshoots are slow to grow, with a small number of roots.
14	0.5	0.3	0.4	1.33 ± 1.15 bcd	11.33%	Microshoots are slow growing, with weak roots.
15	0.5	0.4	0.2	1.00 ± 1.73 bcd	5.67%	Microshoots are poor, with yellowing leaves and a few roots forming.
16	0.5	0.5	0	0.33 ± 0.57 cd	5.67%	Microshoots are poorly grown, with yellowing leaves and healing at the base.

Means followed by the same letters in rows are not significantly different at *p* ≤ 0.05.

**Table 5:** Effects of different substrate proportions on plantlets.

Group	Substrate Ratio	Survival Rate
1	peat soil	0.03 ± 0.05 c
2	peat soil: vermiculite = 1:1	0.26 ± 0.05 b
3	peat soil: vermiculite: perlite = 2:2:1	0.86 ± 0.05 a

Means followed by the same letters in rows are not significantly different at *p* ≤ 0.05.

## Data Availability

All data are available in the manuscript.
